# 1783. Acute Kidney Injury Associations with Antibiotics: A Pharmacovigilance Study of the US FDA Adverse Event Reporting System

**DOI:** 10.1093/ofid/ofad500.1612

**Published:** 2023-11-27

**Authors:** Jose J Kochuparambil, Jomel Raju

**Affiliations:** St. Joseph's College of Pharmacy, Cherthala, Pala, Kerala, India; St. Joseph's College of Pharmacy, Cherthala, Pala, Kerala, India

## Abstract

**Background:**

Antibiotic-associated acute kidney injury is found to be associated with many antibiotics like vancomycin, fluoroquinolones, penicillin combinations, and trimethoprim–sulfamethoxazole. Other antibiotics may also lead to acute kidney injury. Still, no study has systemically compared the cases of acute kidney injury reported to the US FDA Adverse Event Reporting System (FAERS) with the many available antibiotics. Therefore, the study aimed to evaluate the reporting associations between acute kidney injury and different classes of antibiotics through the analysis of the US FDA Adverse Event Reporting System (FAERS).

**Methods:**

FAERS reports from 1 January 2015 to 31 December 2022 were included in the study. The Medical Dictionary for Regulatory Activities (MedDRA) was used to identify AKI cases. Reporting odds ratios (RORs) and corresponding 95% confidence intervals (CIs) for the reporting associations between antibiotics and AKI were calculated. A reporting association was considered statistically significant when the lower limit of the 95% CI was > 1.0.

**Results:**

A total of 2,042,801 reports (including 20,138 AKI reports) were considered. Colistin had the greatest proportion of acute kidney injury reports, representing 25% of all colistin reports. acute kidney injury RORs (95% CI) for antibiotics were, in descending order: colistin 33.10 (21.24–51.56), aminoglycosides 17.41 (14.49–20.90), vancomycin 15.28 (13.82–16.90), trimethoprim–sulfamethoxazole 13.72 (11.94–15.76), penicillin combinations 7.95 (7.09–8.91), clindamycin 6.46 (5.18–8.04), cephalosporins 6.07 (5.23–7.05), daptomycin 6.07 (4.61–7.99), macrolides 3.60 (3.04–4.26), linezolid 3.48 (2.54–4.77), carbapenems 3.31 (2.58–4.25), metronidazole 2.55 (1.94–3.36), tetracyclines 1.73 (1.26–2.36), and fluoroquinolones 1.71 (1.49–1.97).

**Conclusion:**

This study found 14 classes of antibiotics having significant reporting associations with AKI. Among the antibiotics evaluated in this study, colistin had the highest acute kidney injury ROR and moxifloxacin had the lowest.

**Disclosures:**

**All Authors**: No reported disclosures

